# Assessing research participant preferences for receiving study results

**DOI:** 10.1017/cts.2019.427

**Published:** 2019-10-04

**Authors:** Sarah Cook, Stephanie Mayers, Kathryn Goggins, David Schlundt, Kemberlee Bonnet, Neely Williams, Donald Alcendor, Shari Barkin

**Affiliations:** 1 Vanderbilt Institute for Clinical and Translational Research, Vanderbilt University Medical Center, Nashville, TN, USA; 2 Department of Regulatory Affairs, Sarah Cannon, Nashville, TN, USA; 3 Department of Psychology, Vanderbilt University, Nashville, TN, USA; 4 Community Partners’ Network, Nashville, TN, USA; 5 Department of Microbiology and Immunology, Meharry Medical College, Nashville, TN, USA; 6 Department of Pediatrics, Vanderbilt University Medical Center, Nashville, TN, USA

**Keywords:** Dissemination approaches, return of research results, research participants, result dissemination, qualitative methods

## Abstract

**Introduction:**

Dissemination of results to research participants is largely missing from the practices of most researchers. Few resources exist that describe best practices for disseminating information to this important stakeholder group.

**Methods:**

Four focus groups were conducted with a diverse group of individuals. All participants were part of a Patient-Centered Outcomes Research Institute-funded survey study. Focus groups aimed to identify participants’ preferences about receiving research results and their reactions to three different dissemination platforms.

**Results:**

Four focus groups with 37 participants were conducted, including: (1) adults with one comorbidity, at least a college education, and high socioeconomic status (SES); (2) adults with one comorbidity, less than a college education, and lower SES; (3) adults with low health literacy and/or numeracy; and (4) Black or African American adults. Participants discussed their preferences for research results delivery and how each of the platforms met those preferences. This included information needs as they relate to content and scope, including a desire to receive both individual and aggregate results, and study summaries. Email, paper, and website were all popular avenues of presentation. Some desired a written summary, and others preferred videos or visual graphics. Importantly, participants emphasized the desire for having a choice in the timing, frequency, and types of results.

**Conclusion:**

Research participants prefer to receive research results, including study impact and key findings, disseminated to them in an engaging format that allows choice of when and how the information is presented. The results encourage new standards whereby research participants are considered a critical stakeholder group.

## Introduction

Dissemination science is an emerging field that aims to facilitate more effective and strategic application of evidence-based interventions in a variety of settings, including community context. Dissemination is defined by the Patient-Centered Outcomes Research Institute (PCORI) as “the intentional, active process of identifying target audiences and tailoring communication strategies to increase awareness and understanding of evidence and motivate its use in policy, practice and individual choices” [1]. Although organizations such as PCORI [1] and the National Institutes of Health [2] have toolkits to guide dissemination, few specifically address disseminating findings to research participants.

Research participants are a particularly important stakeholder group given that without them, research would not proceed. While research communities generally support returning findings to participants [3–7], this is not a common practice among researchers [6,8]. This may be due in part to limited knowledge about ways to do so, uncertainty about the types of information to return, ethical concerns, financial constraints, or other barriers to dissemination [4,6,8,9].

Research participants have consistently expressed their desire to receive research results back [7,8,10]. Yet, despite calls for action, many questions remain regarding the return of research results to study participants, including how, when, and what types of information should be returned [11]. To begin to address this knowledge gap, we conducted focus groups (FG) comprised of diverse participants who had previously participated in at least one research study in order to identify participant preferences for receiving study results.

## Materials and Methods

### Focus Groups with Research Participants

Four focus groups were conducted with a diverse group of individuals who varied by socioeconomic status, health literacy and numeracy, educational attainment, comorbidity status, and race/ethnicity. All participants were part of a PCORI-funded survey study, had expressed interest in being contacted about additional research opportunities, and lived within 20 miles of Vanderbilt University Medical Center. Potential participants were contacted by email and/or telephone (*N* = 406). A focus group guide was developed to encourage feedback on different representations of data using sample materials and consisted of questions designed to identify participants’ preferences about receiving research results (Supplementary Appendix A). Participants viewed three different platforms for presenting research findings: (1) an animated video that included graphics, text, and background music, (2) a video with a researcher narrating the study findings, and (3) a visual result graph. Focus groups were conducted by two facilitators, audio-recorded, and transcribed by rev.com®. All focus groups took place between May and June of 2018 and lasted for approximately 90 minutes each. Participants received a $20 gift card for their participation.

Informed consent was obtained at the beginning of each focus group. Vanderbilt University’s Institutional Review Board approved all aspects of this study.

### Analysis

**Fig. 1.**
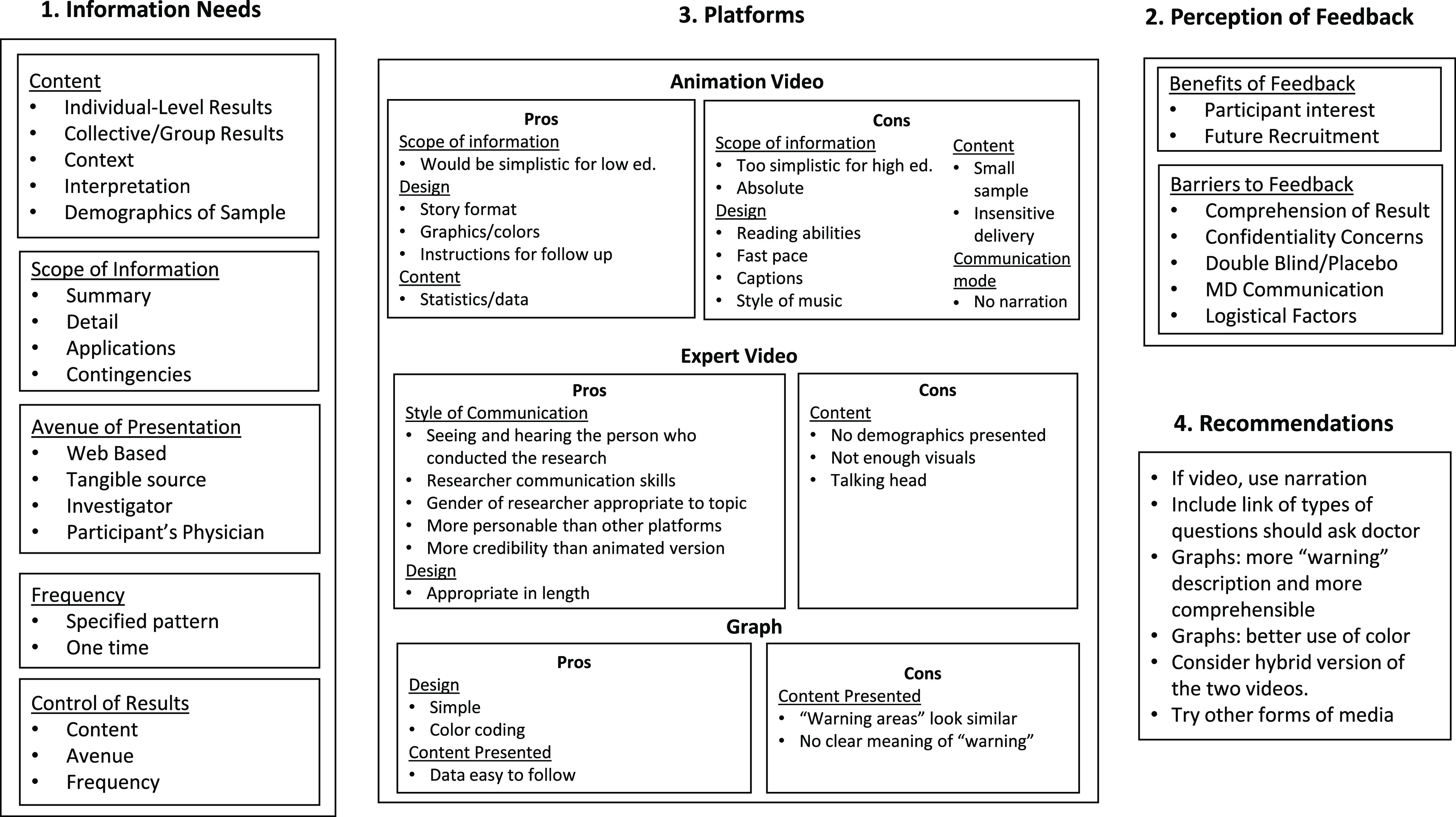
Summary figure – focus group results.

Qualitative data coding and analysis were conducted by the Vanderbilt University Qualitative Research Core and followed the COnsolidated criteria for REporting Qualitative research guidelines, an evidence-based qualitative methodology [12]. A hierarchical coding system was developed and refined using the focus group guide and a preliminary review of two transcripts. The top level of the hierarchical coding system was developed based on the study questions. Additional subcategories were added following a preliminary review of the transcripts. Each major category was subdivided, and the subcategories were further expanded to describe the information related to the study question. This process included both inductive and deductive analysis [13]. Inductively, we used the quotations from the focus groups to identify themes and relationships among themes. Deductively, we were guided by our knowledge of health communication [14] and dissemination theory [15]. All coding was performed by two research assistants who were trained to use the coding system on a selected transcript. Discrepancies in coding were resolved by consensus. The analysis began by reviewing simple frequencies of codes and proceeded by using an iterative inductive/deductive approach to identify themes and connections and develop a conceptual framework (Fig. 1). Management of transcripts, quotations, and codes was done using Microsoft Excel 2016 and SPSS version 26.0 (IBM Corp., Armonk, NY, USA).

## Results

### Focus Group Composition

A total of four focus groups were facilitated with 37 individuals (Table 1). Participant demographics can be found in Table 2. The composition of each focus group varied based on specific recruitment criteria to ensure diversity in opinion and experience was captured.

**Table 1.** Focus group (FG) composition

Focus groups (4)	*N*
FG1: Adults with one comorbidity, at least a college education, and of high socioeconomic status	13
FG2: Adults with one comorbidity, less than a college education, and of lower socioeconomic status	6
FG3: Adults with low health literacy and/or low numeracy	7
FG4*: Adults who identified as Black or African American	11
Total	37

* Recruited based on race/ethnicity; all other FGs were not exclusive to any one race/ethnicity.

**Table 2.** Focus group demographics (N = 37)

Participant characteristics	*N* (%) or mean ± standard deviation
Gender	
Male	19 (51%)
Female	18 (49%)
Race	
White	17 (46%)
Black or African American	17 (46%)
Asian	1 (3%)
Other	2 (5%)
Ethnicity	
Hispanic/Latino	1 (3%)
Not Hispanic/Latino	36 (97%)
Education	
High school	4 (11%)
Some college	6 (16%)
College graduate	13 (35%)
More than college degree	14 (38%)
Average age in years (age range)	56.0 ± 12.6 (range = 27–82)

### Focus Group Findings

We compared frequency of codes across focus groups to ensure that major themes were not specific to a single group. Where relevant, notable between-group differences are highlighted.

Figure 1 captures and organizes participants’ information needs and preferences and the strengths and weaknesses of each platform for meeting those needs, including recommendations for disseminating research findings. In the following sections, we discuss each element of the figure and include exemplar and illustrative quotes from focus group participants.

#### Information needs

##### Content

The first part of each focus group consisted of a general discussion about how and when to receive information from a research study. Two main themes associated with the type of information participants wanted to hear about research results were identified across the four focus groups: (1) individualized information and (2) collective group results. Several participants expressed an interest in a personalized message to help them understand how the findings of the study apply to their specific conditions. “Most people, I think, are really interested in their own situation. ‘How does it affect me?’” (FG1).

Beyond the individual implications, participants expressed interest in general findings and in the ability to compare their results to relevant groups. “Is my A1C higher than theirs? Does it mean I need to do better…?” (FG2). They also wanted a summary that answers basic questions such as what happened, what was learned, and applications of findings.

Providing guidance on further steps or actions that participants should take to promote their health and well-being facilitates meaningful interpretation of the study. “…They could provide people with other resources or at least point them in the right direction … maybe up-and-coming research … A lot of people don’t know what is out there, and what could possibly be available to them” (FG4).

Participants wanted to have the findings broken down by demographic characteristics such as age, sex, race/ethnicity, and neighborhood. This would allow them to know the degree to which the findings of the study apply to people similar to them demographically. “…When they’re doing all of this testing, do they gather by race, color, age, zip codes, all of that? That’s interesting to find out, because I may say, ‘Hey, my zip code, there’s a lot of this going on over here, so we need to start watching that’” (FG4). They were also interested in knowing how well the characteristics of the study sample permit the results to be applied to other contexts.

Furthermore, participants wanted results that have implications for their families and communities now and in the future. “…What do those conclusions mean for us, maybe, or impact us specifically to our health, and how we could possibly use that information in the future” (FG4). Participants would also like to know what direction the researchers are moving toward in future studies.

##### Scope of information

Participants gave some clear guidelines about the kind of information they wanted to receive. A presentation of results needs to have a clear, plain-language summary that lists the key findings. This summary provides an overview and would allow participants to better understand the details that are presented.

In addition to the big picture, some participants also expressed interest in understanding the details. They were especially interested in seeing the data that were used to draw conclusions and recommendations. “I don’t necessarily need to know everything, but I would have loved … the specifics of how they came about that, rather than just the summary of what their conclusions were” (FG4).

Participants also wanted to hear about how study findings apply to health care and policy. “It’s not just what the results of it was, but what impact did it have on future decision-making in healthcare … What did that change?” (FG4). They wanted to know how the study may make life better, and what specific recommendations came from the study. “…What was the action taken after they did the research? Anything that might help us to understand if we had any impact on it or not” (FG4).

Participants in FG1 reflected on some factors that might alter how the feedback is given. One point of discussion was how to handle studies that include placebos, and about whether and how to inform someone that they had been placed in a placebo group. There were also considerations about the timing of the feedback. “…If you’re in the middle of the study or … treatment, you’re going to be a lot more intensely curious about results, given your specific scenario” (FG1). Participants thought that very different information would be given during a study, just after a study, or months to years after study completion.

##### Avenue of presentation

There were several different modes of presentation discussed by participants including web-based or online dissemination, obtaining something tangible like a booklet or handout, seeing a presentation given by a study investigator, and having the results interpreted by a personal physician.

Online results included websites, social media, and communication via email. They thought the value of web-based dissemination included graphic presentations with interactive features. A website with individualized accounts would allow personalized results to be offered securely. Not everyone supported electronic media as a means to disseminate results. Some participants stated a clear preference for obtaining results that are printed on paper. “…Generationally, there are those of us that do prefer to have that hard copy to read the letter in the mail, as opposed to receiving ‘go to the link’” (FG1).

There were some who thought having a presentation from study investigators would be an effective way to share information, allowing participants to ask questions about the findings and to get expert answers. Participants suggested delivering a summary of results before a face-to-face meeting or presentation, in order to prepare questions.

Several participants stated that they would prefer to have study results interpreted by a personal physician. “I think that the doctor should talk to you about the results, give you an opportunity to know what they did find. Because like with my doctor, there’s certain things she suggests” (FG4). This would be a better delivery method for personal findings about health problems. “It depends on what it is. If it’s something bad, you better be where your doctor can get it, because you’re going to have questions” (FG1). This would also allow the results to be individually interpreted and for participants to have their questions answered by someone familiar with their health.

##### Frequency

Participants discussed the frequency and timing of interactions about the study. Many participants thought there would be value in having multiple contacts over time. They believed that the study findings and implications for health might evolve over time and felt they would like to be kept informed. “In the AIDS study … it takes a while for studies to mature. During that period of time … I would want at least quarterly to know where the research stands. What direction is it going?” (FG4). For long-term studies, participants expressed interest in receiving interim findings. “But at least once they figured something out of value, then they should definitely let us know” (FG4). Other participants preferred waiting for feedback until the study is completed and the findings are well understood.

##### Control of results

Participants desired to have control over the content, avenue, and timing of getting study results. One participant suggested having a questionnaire that would lead to the creation of tailored results. Participants preferred to have control over how they receive results, and how often. Several participants wanted the ability to opt in or out of getting specific results, and being able to change the frequency or continuation of receiving results.

#### Perception of feedback

The focus group participants talked about the process of getting feedback and identified a number of benefits and barriers to giving participants feedback about research studies.

##### Benefits

The benefits of giving feedback were grouped into two categories: (1) ways to satisfy people’s interest in the outcome of a study and (2) ways to improve recruitment of participants in future studies. The fulfillment of one’s scientific curiosity was seen as a benefit to receiving research results. Additionally, giving feedback to participants might make them more likely to participate in future research studies and to recommend participation in research to others. “If research centers were better at giving feedback that was interesting and encouraging with the participants, they might be willing to share with their neighbors, 'Hey, this is a great experience! … You ought to participate’” (FG1).

##### Barriers

Compared to benefits, there was much more discussion about the obstacles encountered in sharing research findings with a diverse set of study participants. Communication that is perceived as condescending was seen as a significant barrier to the dissemination of research results. “[Researchers] kind of condescend you. Like, ‘Oh well, this is a little complicated. You may not understand.’… If you truly know what you’re talking about, you are able to teach people or at least give them a good idea of what is going on” (FG4).

Maintaining confidentiality of research results was an important concern. Some pointed to the possibility of potential legal repercussions surrounding the disclosure of private information. Others expressed more practical concerns. “Would you want your kid looking at your email, opening that up, or your co-worker or something. That would be a little rough” (FG2).

Double-blind placebo-controlled studies were seen as more challenging to talk about due to whether investigators should inform individuals about which condition they had been assigned. Some were not sure they would want to know if they had been given a placebo. “If I were in that type of a study, which I’m not, I’m not sure I’d want to know” (FG1).

Participants had concerns about the willingness and ability of researchers to communicate the results of their work. For example, researchers might not be able to disseminate findings until after the study is published, yet after publication, they might be more interested in moving on to something else. They also thought it was difficult for people with advanced degrees, due to lack of training and skills, to create simple communications that could be understood by a range of study participants. “I think there should be a class that PhDs, really anyone in science or thinking about going into research … should take specifically for communicating with people that you’re going to be studying … and methods and ways … to communicate the results with them” (FG4).

Participants also discussed logistical barriers to disseminating results. They saw time constraints as a logistical barrier to having researchers schedule and spend their time giving feedback to study participants. “I understand that the researchers … they already are pressed for time … They have their things they have to write, their classes, all of these things” (FG4). Along with time constraints, funding concerns were also seen as a barrier to disseminating results. “The researcher can’t do it. He ain’t got time to do all the public feedback stuff. So, you’d have to have a grant big enough to hire somebody to do that” (FG1).

#### Platforms

For the second part of each focus group, participants viewed three different platforms for sharing results.

##### Animated video platform

The first platform was an animated video presenting the results of a study on uterine fibroids and chance of miscarriage. Focus groups noted several positive features associated with this platform. Some participants thought it was a good way to package all the necessary findings, including health implications, into a single presentation. “I thought [it] was just an excellent visual representation of some of the complex information, but put forth in a very simplified but accurate way that visually represents that information. You can see the proportions there. So the numbers mean more when you see them in proportion with the little figures, the little icons” (FG4). They thought that the animation would allow for most people to come away with an understanding of the study and its findings. They also thought that follow-up recommendations were beneficial. “Where it said to ‘share this with your doctor,’ it puts a lot more value on it” (FG1).

Participants liked the design of the animation, especially the fact that it allowed the study findings to be presented as a narrative. The use of animation allowed the incorporation of graphics and colors and made the results pleasing to see. “I’m a visual person … I have to see it” (FG4).

Participants also identified a range of things they disliked about the animation. They saw the animation as having limitations as far as the scope of information covered. Some thought that the information was presented in a way that was too absolute. “In your one limited study, with a rather small sample size … that made me cringe, honestly, as to how solidly they were presenting their findings outside of a wider context” (FG1). Others saw it as being oversimplified, especially for participants with higher education. “My scientific training – there were a few other details in there I would have liked to have seen that I don’t think, necessarily, would have been useful to a wider audience” (FG1).

There was a range of criticisms about the design of the animation. The animation relied on images and text with background music. Some thought it moved too fast. Some mentioned that keeping up with reading the text was difficult and would be especially difficult for people with limited literacy skills. Participants also mentioned that animation is inappropriate for a personal topic, such as uterine fibroids. “…It’s terribly annoying. And really for the topic that we’re talking about, it’s a little bit disrespectful” (FG3). There was much dissatisfaction with the use of text alone, with no verbal narrative in the animation.

##### Expert video platform

The second platform was a video recording of the research physician explaining the results of the study. Participants’ commentary was mostly positive, seeing it as authoritative and informative. Participants thought it was more thorough than the first animated video – “[This video] … was everything I felt was missing from the first one” (FG1) – and that it contained more credible scientific findings. “To me, the [expert video] was more scientific. Even [though the animated] one showed the statistics” (FG1).

The participants felt that the researcher’s presentation was a good way to share the study results. “It makes it more personal, more human and less clinical” (FG4). Seeing the actual researcher, putting a name and face to the study, and sensing the enthusiasm and excitement the researcher had about the study were all communicated through the expert video. Participants thought that this version allowed the viewer to better understand the context in which the research was done, had accurate and useful information, and gave insight into the researcher’s motivations for doing the study. “She explained why she did the research … and the reasoning, was great, because then you have an explanation of why she did it. Then for the [participant], you actually see, ‘okay, this is why she had us do this study’” (FG4).

Some participants felt better informed and more reassured after watching the expert video compared to the animation. “I saw reassurance from the presenter herself because of the expressions on her face … that reassurance that I think would go to the person that’s looking at the video, who would have fibroids” (FG3). Because the study was about gynecologic problems, the participants appreciated that the speaker was a woman, as well as a surgeon. “I think some of the glue to her effectiveness is that she came across as believable and empathetic and kind. And, if you had some 6’6” burly, gruff guy, saying the exact same thing, it would have been a totally different thing” (FG1).

There were some unfavorable reactions to both the content and the design of the expert video. Some wanted more information and a graphic presentation of the data. One participant found the expert video boring compared to the animation, while another saw the message being conveyed as different from the one portrayed in the animation. Another thought that keeping the camera on the speaker during the entire video detracted from the ability to provide easy to understand information about the study. “The camera was on her talking the entire time. And I liked that at the beginning because it built credibility and a relationship. But I would have been okay if we bounced away from that…” (FG3).

##### Graph

The third platform was a graph. The graph had strengths and weaknesses. Positively, the design of the graph contributed to better interpretation of the findings. One participant liked the way the graph could be used to represent a lot of information at the same time. “…So I can kind of see and figure out if there are any patterns or any trends, and how that kind of plays into whatever question I’m trying to answer” (FG4). There was also positive discussion about the use of color as a way to convey meaning in the graphs. “The color coding very much makes it easier to see where you fit” (FG1).

There were also negative comments about the graph. These had to do with ambiguities and the need for more specific labeling or a verbal explanation to go along with the graph. “It just says ‘warning’, ‘warning’, and ‘trouble’ … The labeling is not clear…” (FG2). Several participants described difficulties they had in reading and interpreting graphs, including the need for a narrative to explain the areas and terms used on the graphs.

The graph’s color-coding may have violated participants’ expectations due to stereotypes of what different colors mean. “…Red. That means a signature or a ‘Code-Red’. We know red as dangerous. When… ‘in the red’, we’re doing badly. We attach lots of colors for how we perceive them …” (FG3). One participant just clearly stated that s/he did not like graphs.

#### Recommendations

The participants made a number of recommendations for improving the quality and usefulness of the materials they had viewed during the focus groups. Most of these were suggestions to add more material or details to the study results. They wanted to have information in the results that would satisfy both those who desire an overview and those who appreciate more detailed information. This included a suggestion to prepare a participant with questions to ask their doctor. “It (animated video) said in there to ask questions of your doctor. It might be nice if it had a link or some way to get to what questions do you ask” (FG1).

There were also suggestions to be open to different presentation styles and to have rich content that would speak to people with different learning styles and needs. “…There are different kinds of learners. There are auditory learners. There are visual learners. The first (animated video) lent itself to a multiple number of learners, where the second (expert) video, if you were an auditory learner you were good, because you understood, because she did a lot of talking. But if you were a visual learner, you were kind of out of luck simply because the second one did not give you the opportunity to see” (FG4).

The animation could have been improved by using a voice-over in addition to the written text. “…I thought [the animated video] was very effectively done … but I would’ve also appreciated the narration … I’d want to hear it, not just sit and watch it” (FG2). One participant suggested creating a podcast to accommodate auditory learners.

Rather than preferring only one of the three presentation styles reviewed in the focus groups, some participants specifically stated that they thought a hybrid presentation that combined the best of each of the approaches would be more effective. “If you’ve ever been to TEDx … and watched the TED Talks, the multimedia presentations are most effective. So, they’re using a combination of statistics, video, just mixing it up” (FG1). They were also open to having a lengthier hybrid presentation.

## Discussion

Consistent with previous studies, our findings indicate that participants’ desire to receive results from the research studies they participate in. However, a “one-size-fits-all” dissemination approach does not apply – rather, a multipronged approach is required to meet the diverse needs and preferences of study participants. For example, focus group participants wanted information that was relevant to their needs and priorities, including individualized findings. They desired both a concise summary along with supporting details. Study results should summarize overall findings, while providing enough detail to allow participants to compare themselves to others. When creating content and materials to disseminate, researchers should draw on principles from health literacy, numeracy, and communication.

Our findings align with the literature, which has demonstrated research participants’ desire to receive both aggregate and clinically significant individual study results [8,16]. Participants wanted to know the study’s implications and how the research might change the practice of medicine or advance medical treatment. When returning study findings, it may be important to include these elements to satisfy a wide range of people. However, ethical implications must be considered if sensitive information is to be returned individually [5,17–20]. Specifically, some participants endorsed having a personal physician deliver individualized study results as a better method for receiving sensitive or negative clinical research findings, an approach that has been previously recommended [7,21].

Given that focus group participants were open to a variety of pathways and platforms for receiving study findings – from personalized interactive online web applications, to mailed paper copies of study results – a multimodal strategy should be considered when disseminating research findings to study participants. This includes visuals that are carefully tailored to the study type, health condition, and target population. Specifically, some focus group participants desired a hybrid video approach. They liked seeing and hearing “the science” and being presented with data, but also wanted a “human aspect” to the research – hearing the researcher speak and provide context to the study and findings. This hybrid approach has the benefit of catering to a range of learning styles.

People wanted to have control over how, when, and how often they receive study results. They wanted the opportunity to adjust the frequency and timing during the course of a longitudinal study. Timing of dissemination is an essential point of consideration for researchers [9], both in determining how to meet the dissemination needs of study participants, while also maintaining the scientific integrity of their research. There may be a disconnect with how participants see the timing of study results versus what researchers can reasonably provide. This issue points to the need to communicate early and clearly throughout the study and assess participant expectations. Future research could test the utility of offering participants’ choice in the timing and types of information they receive, perhaps by developing modules that target different preferences at various time points.

While trust was not specifically addressed in the focus groups, some participants mentioned that returning study findings could increase one’s likelihood to recommend participating in research. Given the multitude of well-known, documented disparities in research participation [22–27], disseminating research results to participants could be one meaningful way to build trust and encourage future research participation [6,9,10,28,29]. Future research should assess how returning study findings impacts participants’ trust and likelihood of participating in future research studies. Additional work could explore how communities and the general public are impacted by returning research findings to participants.

Communicating results clearly and without condescending tones are skills that researchers should master. Researchers could address these concerns by: (1) proactively working to improve their communication and teaching skills and (2) seeking out resources to assist with articulating their study’s findings to a range of literacies, educational attainments, cultural backgrounds, and experiences. Researchers are encouraged to partner with others – either individuals, departments, or organizations – who have experience and expertise in dissemination, health communication, and health literacy to assist with the dissemination of their study findings. Future work could focus on developing resources and training for researchers that provide the tools and strategies they need for effectively communicating and disseminating study findings back to research participants.

A second approach could be a core service with whom researchers would work when planning their dissemination to broad audiences. In this scenario, research institutions would have a communication core the way they have other biomedical cores, elevating the need and expectation for clear communication of research findings for all audiences who could benefit from learning the results.

## Limitations

We acknowledge a few limitations of our methods and results. First, our focus group participants had a relatively high level of educational attainment and did not include a large number of individuals who identified as Asian or Hispanic. All participants were English-speaking, so we cannot generalize findings to non-English speakers. Additional work should be done to glean the perspectives and opinions of other sociodemographic and historically underrepresented groups in research. Second, while some of the participants had taken part in other research studies, all were recruited from a single survey study. Future research should recruit participants who have taken part in different types of research, such as longitudinal studies and clinical trials, and in a variety of topic areas in order to include their preferences on the return of research results.

## Conclusion

Very few researchers disseminate research results to study participants, and resources describing best practices for research participant dissemination are scarce. There is a strong need for researchers to develop and implement strategies for returning research findings back to participants based on best practices and recommendations. While current research standards include identifying a dissemination plan, there is no specific mention that dissemination needs to include research participants. The results of our work encourage us to set a new standard, whereby research participants are considered a critical stakeholder group. Our findings provide a promising starting point for beginning to address this gap in knowledge. Researchers should return study findings to their research participants and consider these specific recommendations to help guide their dissemination efforts, materials, and budget. Funding agencies should simultaneously encourage the development of materials and dissemination of research findings back to study participants.

## Supplementary material

For supplementary material accompanying this paper visit https://doi.org/10.1017/cts.2019.427. click here to view supplementary material
