# Supplementary material for: Assessing research participant preferences for receiving study results
Source: J Clin Transl Sci. 2019 Oct 4;4(3):243–9. doi: 10.1017/cts.2019.427 (PMC7348009; doi:10.1017/cts.2019.427)

**Appendices**

**Appendix A.** These were the questions developed by the Vanderbilt team that were administered during the focus groups with research participants.

**Focus Group Facilitation Guide**

1. What types of information have you received after participating in a research study?
   1. Who provided you with the information?
   2. What did you think about this information?
      1. Was it helpful/interesting?
      2. What else would you have liked to receive?
2. What types of information would you like to receive after participating in research?
3. How should this information be presented to you?
4. Who should receive this information (family, doctors, etc.)?
5. How soon would you like to receive the information?
6. What platform should be used to convey information?
7. What information would you like to receive after this focus group?

Participants were shown three examples of research results and asked to comment on which one they preferred:

1. animated video: <https://www.youtube.com/watch?v=qm3zd5vUBRw>
2. video with in-person researcher narrating research findings: <https://www.youtube.com/watch?v=SOV5TbnR0DU>
3. graph of individual results


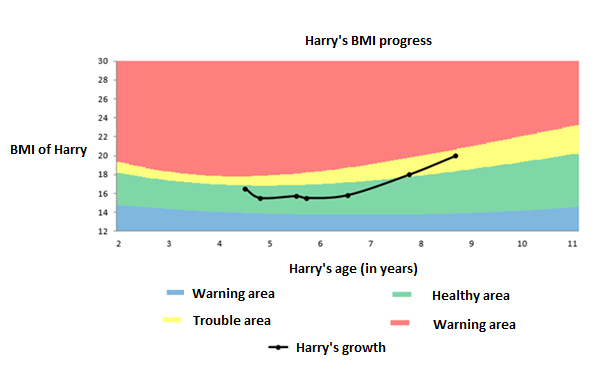

Supplement: Supplementary file 1 [file S2059866119004278sup001.docx]
